# The Manufacturing and Characterisation of Eugenol-Enclosed Liposomes Produced by Microfluidic Method

**DOI:** 10.3390/foods12152940

**Published:** 2023-08-03

**Authors:** Jessica Ghodke, Sotirios I. Ekonomou, Edward Weaver, Dimitrios Lamprou, Olena Doran, Alexandros Ch. Stratakos

**Affiliations:** 1 College of Health, Science and Society, University of the West of England, Coldharbour Ln, Bristol BS16 1QY, UK; jess.ghodke@uwe.ac.uk (J.G.); sotirios.oikonomou@uwe.ac.uk (S.I.E.); olena.doran@uwe.ac.uk (O.D.); 2 School of Pharmacy, Queen’s University Belfast, 97 Lisburn Road, Belfast BT9 7BL, UK; eweaver01@qub.ac.uk (E.W.); d.lamprou@qub.ac.uk (D.L.)

**Keywords:** liposomes, microfluidics, eugenol, storage stability, food safety, emerging technologies

## Abstract

In this study, liposomes enclosing eugenol were prepared using microfluidics. Two lipids—1,2-dimyristoyl-*sn*-glycero-3-phosphocholine, 18:0 (DSPC) and 2-dimyristoyl-*sn*-glycero-3-phosphocholine, 14:0 (DMPC)—and microfluidic chips with serpentine and Y-shaped micromixing designs were used for the liposomal formulation. Minimum bactericidal concentration (MBC) values indicated that eugenol was more effective against Gram-negative than Gram-positive bacteria. Four different flow-rate ratios (FRR 2:1, 3:1, 4:1, 5:1) were explored. All liposomes’ encapsulation efficiency (EE) was determined: 94.34% for DSPC 3:1 and 78.63% for DMPC 5:1. The highest eugenol release of 99.86% was observed at pH 4, DMPC 3:1 (Y-shaped chip). Liposomes were physically stable at 4, 20 and 37 °C for 60 days as determined by their size, polydispersity index (PDI) and zeta potential (ZP). The most stable liposomes were observed at FRR 5:1 for DSPC. EE, stability, and eugenol release studies proved that the liposomal formulations produced can be used as delivery vehicles to increase food safety.

## 1. Introduction

Foodborne illnesses are among the highest risk factors for mortality worldwide. According to the World Health Organization (WHO), approximately 10% of the world’s population, or 600 million people, become ill from contaminated food annually, leading to 420,000 deaths [1]. The availability of safe food is essential for good health and human safety, overall increasing public health. Consumers are becoming more aware of the health hazards associated with chemical preservatives, and the demand for green, safe food with natural preservatives is increasing. Natural compounds of plant origin derived from essential oils (EOs) have gained attention in improving food safety because of their antimicrobial activity, antioxidant, anti-inflammatory and analgesic properties [2]. EOs consist of low molecular bioactive substances and can be used as an alternative strategy for suppressing the growth of pathogens that commonly contaminate food [3].

Eugenol (4-allyl-2-methoxy phenol) is a phenylpropanoid with an allyl chain-substituted guaiacol. It is a naturally occurring phenol present in clove, basil, bark and leaves of cinnamon, and lemongrass. It shows broad-spectrum antimicrobial activity against Gram-negative (e.g., *Pseudomonas aeruginosa*, *Salmonella typhi*, *Yersinia enterocolitica*, *Helicobacter pylori*, and *Escherichia coli*) and Gram-positive bacteria (e.g., *Streptococcus pneumoniae*, *Staphylococcus aureus*, *Enterococcus faecalis*, and *Streptococcus pyogenes*) [4]. Eugenol is approved for use in food and is a Generally Recognised as a Safe (GRAS) food additive by the U.S. Food and Drug Administration (FDA) [3]. Incorporating eugenol in lettuce juice and minced pork was found to exert an antimicrobial effect on *Shigella flexneri*. A significant reduction of 5.52 and 6.60 log CFU/mL was observed in lettuce juice and minced pork, respectively, after using eugenol [5].

On the other hand, the use of eugenol, like other EOs, has a number of limitations related to its poor solubility, susceptibility to oxidation, and hydrolysis after exposure to high temperatures during food processing leading to its denaturation and loss of function. This can be prevented by encapsulating eugenol into nanostructures such as liposomes, which are spherical vesicles made up of lipids. Liposomes are ideal candidates for the encapsulation of different molecules due to desirable attributes such as non-toxicity, biodegradability, and the capacity to facilitate sustained release of the enclosed compound [6]. Their unique structure allows the encapsulation of hydrophilic compounds in the central aqueous cavity, while hydrophobic compounds are embedded in the lipid bilayer. Enclosing bioactive compounds within liposomes protects them from external environments, enhancing the stability and bioavailability of the compound without compromising its effectiveness [6,7].

Liposomes can be produced using several methods, and technological advancement has paved the way for sophisticated high-throughput methods such as microfluidics. The microfluidic system enables precise control over the mixing ratio of the aqueous solvent and ethanol, impacting the liposomal formulation’s particle size and polydispersity. This method is faster and more economical than other methods due to the use of low volumes of solvents and time efficiency. Furthermore, microfluidics allows the use of microfluidic chips with different micromixing patterns that can be explored to obtain liposomes with varying physical parameters. The difference in the design of micromixing channels is reported to influence the mixing of the ethanol and aqueous phase, thereby affecting the size and polydispersity of the liposomal formulation [8].

Considering these factors, microfluidics can be regarded as a sustainable method for liposome production. It facilitates the production of liposomes in a range of 50–250 nm with low polydispersity and prevents the need for post-production downstream treatment. In addition, software programs aid in controlling various parameters, enabling reproducible results [9].

In the last decade, liposomes have been used to stabilise food components, fortify dietary supplements, and encapsulate antimicrobial compounds [6]. Numerous studies show that the antimicrobial compounds enclosed in liposomes exhibit higher antimicrobial activity when applied to food products compared to their free form as non-encapsulated compounds [10,11]. Thus, this has led to an increased demand for liposomes that can maintain the functional properties of the enclosed compounds and improve the food products’ safety.

To the best of the authors’ knowledge, there are no studies on the production of liposomes enclosing pure eugenol compound using microfluidics. Therefore, this study aimed to produce eugenol-enclosed liposomes using microfluidics to inhibit or reduce the growth of commonly occurring food pathogens (e.g., *E. coli* and *S. aureus*) to increase food safety and protect public health. A wide range of manufacturing parameters was investigated to identify the best parameters for liposome production. The specific focus was on the use of four different flow-rate ratios (FRR; 2:1, 3:1, 4:1, and 5:1), two microfluidic chips with different micromixing channel designs (e.g., serpentine and Y-shaped), and two lipids (e.g., DSPC and DMPC). The encapsulation efficiency (EE) of the liposomal formulations was determined to ascertain the amount of eugenol enclosed. Following this, the stability of the eugenol-encapsulated liposomes was investigated by extensive characterisation of their physiochemical parameters, such as size, PDI, and zeta potential (ZP), throughout a storage period of 60 days. In vitro drug release studies were undertaken to elucidate the eugenol release from liposomes at common food pH values of 4 and 7.4.

## 2. Materials and Methods

### 2.1. Preparation of Bacterial Strains

Two bacteria strains of *E. coli* (ATCC 25922 and NCTC Tox-12900) and one *S. aureus* strain (NCTC 12981) were revived from a frozen stock stored maintained at −80 °C. A single bead was transferred to 10 mL Mueller–Hinton broth (MHB) and incubated overnight at 37 °C. A loopful of each strain was plated on sterile tryptic soy agar (TSA) and incubated overnight at 37 °C. A single colony of each strain was inoculated in 10 mL MHB and incubated overnight at 37 °C for 24 h. To prepare the working culture, the broth was centrifuged at 6500× *g* at 4 °C for 15 min. The cell pellet was resuspended in maximum recovery diluent (MRD) and further diluted in phosphate-buffered saline (PBS) to obtain the final concentration of 10^6^ colony-forming units/mL.

### 2.2. Determination of Minimum Inhibitory Concentration (MIC) and Minimum Bactericidal Concentration (MBC)

The MIC value of eugenol for *E. coli* ATCC 25922, *E. coli* NCTC Tox-12900 and *S. aureus* NCTC 12981 was determined by broth macrodilution according to Clinical and Laboratory Standards Institute (CLSI) protocol [12]. Eugenol dilutions in the range of 0.1–8.0 mg/mL were prepared in sterile 10 mL Falcon tubes filled with MHB to 10 mL after adding the appropriate amount of eugenol and bacterial inoculum. A 24 h culture of each strain (100 µL, approximately 10^6^ CFU/mL) was used as inoculum. Triplicates of each concentration, along with negative and positive controls, were made. After this, the tubes were incubated at 37 °C and checked for turbidity after 24 h. MIC was considered the concentration that prevented the visible growth of microorganisms, and no turbidity was observed in the tubes. For MBC determination, 100 µL from the tubes with no turbidity was spread plated on TSA and incubated at 37 °C for 24 h. MBC was considered the concentration where no bacterial growth was observed on the TSA plates.

### 2.3. Preparation of Liposomes

Liposomes were synthesised using the Microfluidic (MF) LineUp Push-Pull pressure-controlled system (Fluigent, Paris, France). Two different phospholipids—2-dimyristoyl-*sn*-glycero-3-phosphocholine, 14:0 (DMPC) and 1,2-dimyristoyl-*sn*-glycero-3-phosphocholine, 18:0 (DSPC)—were used for the preparation of the liposomes and were purchased from Avanti Polar Lipids (Alabaster, AL, USA). The microfluidic system consisted of two reservoirs: one was filled with PBS (pH 7.4) and the second with solvent solution (99.8% pure ethanol (Merck, London, UK)), containing dissolved lipid and cholesterol at a ratio of 2:1 [7] and eugenol at MBC concentration 0.4 mg/mL. The pressurised fluids from the reservoirs flowed through separate inlet channels to a microfluidic chip. Two microfluidic chips with different micromixing channel designs (serpentine and Y-shaped) were used. An image of the microfluidic chips is provided in Supplementary Material Figure S1. Liposomes were prepared at different flow-rate ratios (FRRs) of the aqueous (PBS)-to-ethanol phase and were adjusted to 2:1, 3:1, 4:1, and 5:1 ratios. The total flow-rate ratio was maintained at 500 µL/mL.

### 2.4. Stability Study of Liposomes

To study the stability of liposomes, physiochemical parameters such as particle size, zeta potential (ZP) and polydispersity index (PDI) of liposomes were characterised during storage on days 0, 14, 28, and 60. The liposomal formulations were stored at 4, 20, and 37 °C. Particle size, PDI and ZP were measured on days 0, 14, 28, and 60. A suspension of 10 µL was diluted with 990 µL PBS and filtered through a 20 μm Millipore filter (Merck, Darmstadt, Germany). The mean diameter size and the size distribution of filtered samples were measured by dynamic light scattering (DLS) using a Zetasizer Nano series ZS (Malvern Instruments Ltd., Malvern, UK) in a 1 mL disposable cuvette (Merck, UK). The folded capillary zeta cell (Malvern Panalytical, Tokyo, Japan, DE) was used for measuring ZP. The ZP was determined by measuring the direction and velocity of the liposomes’ movement in the applied electric field. All the physiochemical parameters of liposomes were measured at a fixed scattering angle of 137° [6,13].

### 2.5. Fourier-Transform Infrared Spectroscopy (FT-IR) Analysis

DMPC and DSPC liposomes were prepared and analysed using the (ATR)-FTIR spectrometer (Nicolet 50, Thermo Fisher Scientific, Oxford, UK) with a built-in ATR. The samples were prepared as described in our previous paper [6] by centrifugation at 1100× *g* for 30 min at 25 °C. After the centrifugation, the supernatant was removed and only the pellets were used in the analysis. Scans were performed over a wavelength range of 4000–650 cm^−1^, with 32 scans per sample at a resolution of 4 cm^−1^ and an interval of 1 cm^−1^. All samples were tested in triplicate on the preparation day (day 0) to ensure that the liposomal formulations had not degraded. Finally, the background absorption was subtracted from the analysis.

### 2.6. Atomic Force Microscopy (AFM) Analysis

A TT-2 AFM (AFM Workshop, Signal Hill, CA, USA) was used to perform the AFM analysis and assist with visualising and characterising the liposomal formulations. A volume of 10 μL from each liposomal formulation was mixed with PBS until a final volume of 2 mL. Fifteen (15) microliters of this dilution were then pipetted onto a freshly cleaved mica surface (1.5 cm × 1.5 cm; G250–2 mica sheets 1″ × 1″ × 0.006″, Agar Scientific Ltd., Essex, UK). The samples were washed with 1 mL PBS to remove the loosely settled liposomes from the mica sheets. Then, the samples were left to dry for 30 min before scanning under ambient conditions. The ohm (Ω) cm antimony-doped Si probes (frequency = 167 kHz) were used to image the samples at a scan rate of 0.6 Hz and 512 × 512-pixel resolution over an area of 5 μm.

### 2.7. Determination of Encapsulation Efficiency (EE)

Once the liposomes were synthesised, 1 mL of each formulation was transferred to sterile Eppendorf tubes and centrifuged at 11,000× *g* for 20 min at 20 °C. The supernatant was collected to determine the encapsulation efficiency. The concentration of eugenol in the supernatant was determined using a UV-vis spectrophotometer (GENESYS 150, Thermo Scientific, UK). Standard eugenol solution was analysed in the 200–300 nm UV wavelength range, and the maximum absorption wavelength of eugenol was 211 nm. A calibration curve was produced and revealed a linear relationship between the concentration of eugenol (x) and the UV absorbance (y). The calibration curve was used to calculate the EE of the liposomes [6]. The final concentration (%) of the encapsulated compound was calculated according to Equation (1):

%EE = (IC − SC)/IC × 100(1)

where IC and SC represent the initial and the supernatant concentration of eugenol, respectively.

### 2.8. In Vitro Drug Release from Eugenol-Loaded Liposomes at Different pH

The liposomal formulations were centrifuged at 11,000× *g* for 20 min to study the drug release. Then, the supernatant was discarded, and the pellet was washed with distilled water. The centrifugation and washing steps were repeated twice to ensure the liposomal preparation was free of eugenol. A cellulose tubing membrane (MWCO; Sigma, Darmstadt, Germany) with a width of 10 mm and 14,000 Da molecular weight cut-off was used. The membrane was cut into appropriate sizes, rinsed with distilled water, boiled for 30 min, and rinsed with distilled water again before use. Drug release studies were carried out at pH 7.4 and 4.0. For pH 7.4, liposomes were suspended in 1 mL of PBS and in the case of pH 4.0, the liposomes were suspended in sodium citrate buffer. One end of the membrane was tied, and the liposome formulation was transferred to the membrane tube. After the transfer, the open end was tied, and the membrane was transferred to a tube containing 9 mL of PBS in case of pH 7.4 and 9 mL of sodium citrate buffer for pH 4.0.

Eugenol release was studied for 7 days. It was monitored every 60 min for 3 h on day 0 and then after 24, 48, 72, 120, and 168 h. At each time point, a sample of 500 µL was removed from the tube and replaced with 500 µL of PBS pre-equilibrated at 37 °C (for pH 7.4) and with sodium citrate buffer in the case of pH 4.0 to maintain constant volume. Released eugenol was determined by reading the sample’s absorbance at 211 nm. The concentration of eugenol was determined from the standard calibration curve. The final drug release from liposomes at both pH values was calculated according to Equation (2):

% RR = SC/IC × 100 (2)

where RR is the release rate of encapsulated eugenol in the liposomal formulations, while IC and SC correspond to the initial and the supernatant concentration of eugenol, respectively.

### 2.9. Statistical Analysis

All the experiments were repeated in triplicate, and the data are expressed as means ± standard deviation (SD). Statistical analysis was conducted by one-way analysis of the variance (ANOVA) along with Tukey’s post hoc test and Student’s *T*-test. The differences were viewed as statistically significant with *p*-values of less than 0.05. All calculations were performed using Minitab 21.1 software.

## 3. Results and Discussion

### 3.1. MIC and MBC Determination of Eugenol

Eugenol showed antimicrobial activity against all the microorganisms tested. The MIC value of eugenol for *E. coli* ATCC 25922 and *E. coli* NCTC Tox-12900 was 0.015 mg/mL, while for *S. aureus* NCTC 12981, it was 0.010 mg/mL. MBC values of eugenol for *E. coli* ATCC 25922 and *E. coli* NCTC Tox-12900 were 0.10 mg/mL, and for *S. aureus* NCTC 12981, it was found to be 0.40 mg/mL.

Eugenol is known to exert its antimicrobial action by disrupting the bacterial cell membrane, leading to the leakage of cell content and disturbance of integral membrane proteins and processes, including ATP synthase-dependent energy generation [14]. In this study, eugenol was more effective against Gram-negative bacteria than Gram-positive, indicated by the lower MIC and MBC values for the strains of *E. coli*. This higher antimicrobial activity against Gram-negative bacteria can be attributed to the hydrophobic nature of the eugenol, which facilitates its passage through the lipopolysaccharide membrane and then across the cytoplasmic membrane [14]. Similar results were reported by Silva et al. [15], who observed lower MIC and MBC values of eugenol in the case of *E. coli* when compared to *S. aureus*.

### 3.2. Preparation of Liposomes

**Figure 1:**
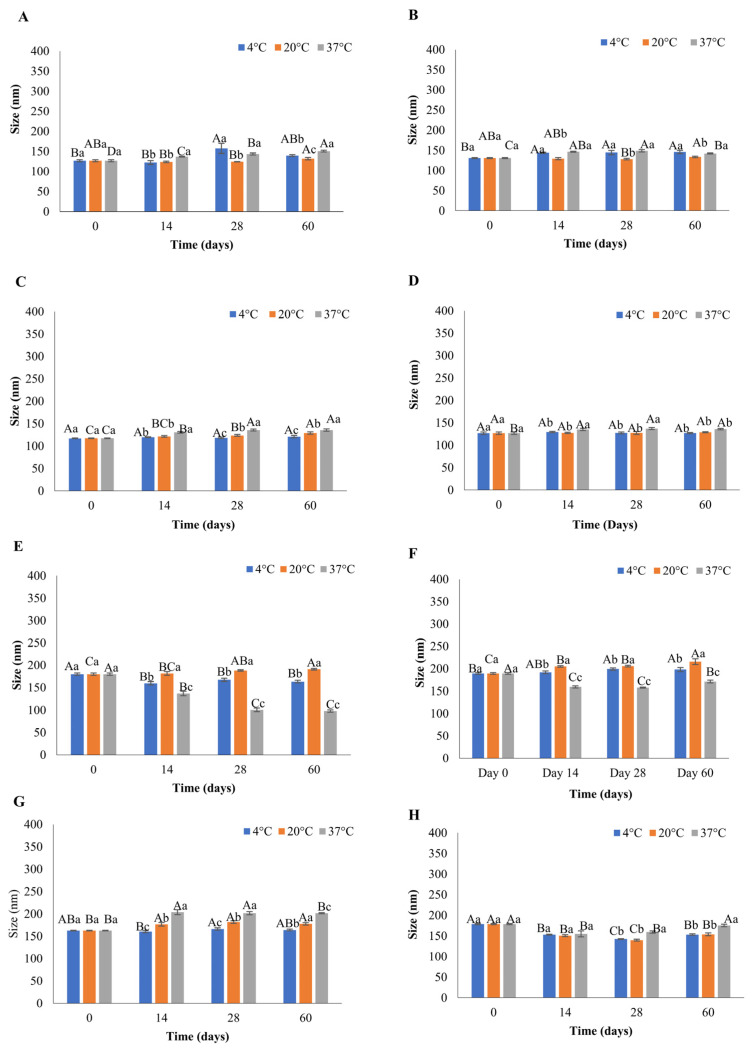
Effect of storage time and temperature on the size of eugenol-loaded liposomes during storage, produced with serpentine microfluidic chip: (**A**) DSPC FRR2:1; (**B**) DSPC 3:1; (**C**) DSPC 4:1; (**D**) DSPC 5:1; (**E**) DMPC 2:1; (**F**) DMPC 3:1; (**G**) DMPC 4:1; (**H**) DMPC 5:1. Different uppercase letters denote the difference in the size of liposomes for a single temperature on different days. Different lowercase letters denote the difference in the size of liposomes at different temperatures on the same day.

**Figure 2:**
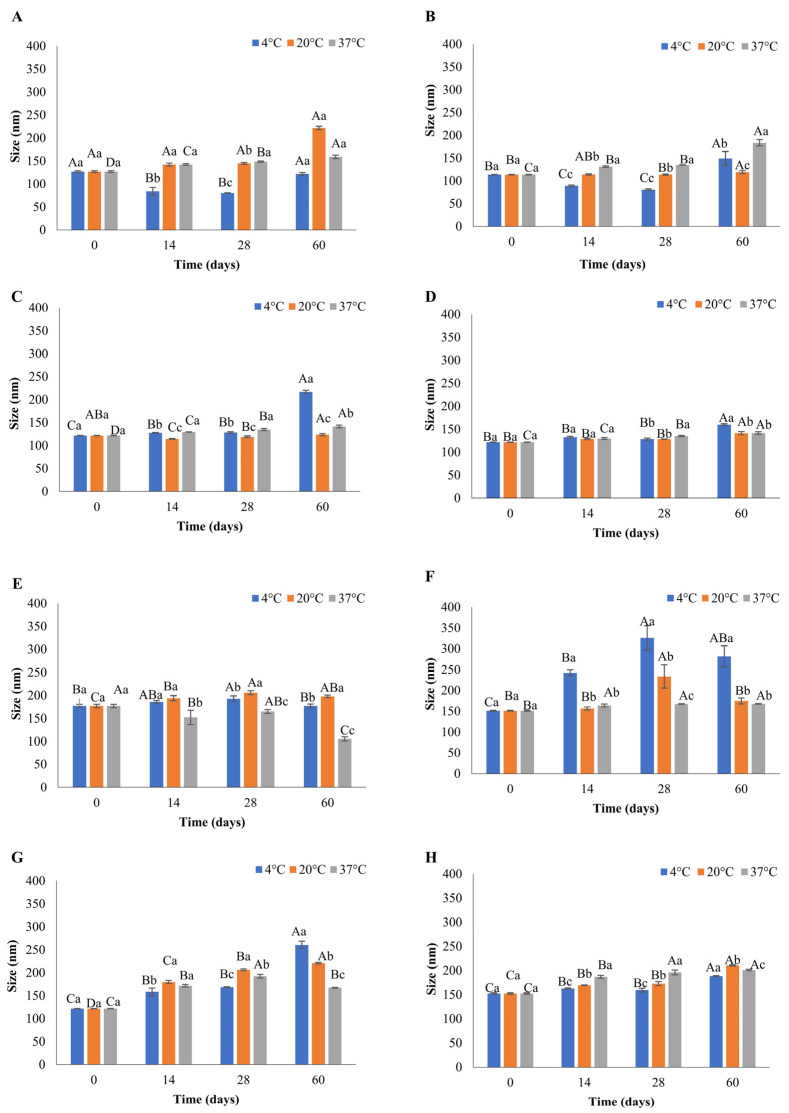
Effect of storage time and temperature on the size of eugenol-loaded liposomes during storage, produced with Y-shaped microfluidic chip: (**A**) DSPC FRR2:1; (**B**) DSPC 3:1; (**C**) DSPC 4:1; (**D**) DSPC 5:1; (**E**) DMPC 2:1; (**F**) DMPC 3:1; (**G**) DMPC 4:1; (**H**) Y DMPC 5:1. Different uppercase letters denote the difference in the size of liposomes for a single temperature on different days. Different lowercase letters denote the difference in the size of liposomes at different temperatures on the same day.

When preparing liposomes for food or pharmaceutical applications, physical characteristics, including size and PDI, are critical, as these govern the enclosed product function, stability during processing and appearance in terms of the final product’s colour. Liposomes smaller than 300 nm appear clear, making them ideal for use in food products [16]. Manipulation of flow-rate ratios of the aqueous ethanol solvent is a key parameter of microfluidics that allows regulation of the size and PDI. This facilitates the production of smaller liposomes than other conventional methods [9]. The present study demonstrated that the size of the liposomes prepared using microfluidics on day 0 was between 113.33 nm and 189.26 nm, as presented in Figure 1 and Figure 2. This is consistent with the data of Shah et al. [17], who showed that the size of the liposomes produced with the microfluidic approach is in the range of 100–200 nm and is significantly smaller when compared to eugenol liposomes prepared with hot homogenisation (332 nm) [18].

Small and homogeneous liposomes are crucial for application in the food industry. Therefore, four different FRRs (2:1, 3:1, 4:1, and 5:1) were investigated and used for the preparation of liposomes. The smallest of liposomes were observed in the case of DSPC FRR 3:1 using the Y-shaped chip, possessing a mean size of 113.33 nm (Figure 2B) with a PDI value of 0.119 (Figure S3B). Likewise, amongst the liposomes produced using the serpentine chip, DSPC FRR 4:1 were the smallest, displaying a mean size of 117.50 (Figure 1C) with a PDI value of 0.104 (Figure S2C). The current study’s findings are consistent with the results of Kastner et al. [9], who reported that increasing the FRR is associated with decreased size of liposomes. Numerous studies reported a similar relationship between the size of liposomes and FRR, but in these cases, the FRRs used were higher than in the present study [19,20]. It should be noted that in the present study, the differences in FRRs on day 0 resulted in small but significantly different (*p* < 0.05) variations in the size of the liposomes (Figure 1 and Figure 2), indicating the importance of maintaining the control of FRRs during the liposomal formulation. Despite the small size variations, all the liposomal formulations produced in the current study were below 200 nm in size. The particle size distribution of liposomes is described by the PDI value, which can be in the range between 0.0 (a uniform sample) and 1.0 (a sample with varied size or multiple size patterns) [16]. Liposomes prepared in the current study had PDI values ranging from 0.09 to 0.24 on day 0, signifying a monodispersed liposomal formulation (Figures S2 and S3). A positive relationship between FRR and PDI values was observed, which is consistent with the results reported by other studies [9,21].

ZP is a measure of the surface charge present on the nanoparticle. Depending on various factors, it can be anionic, cationic, or neutral [22]. The ZP of the liposomes obtained in the present study ranged from −16.43 to −24.20 mV. This study did not reveal any significant effect of different FRRs on ZP of the liposomes, irrespective of which lipids were used for liposome formulation. Similar results were reported by Guimarães Sá Correia et al. [13], who did not observe any significant difference in ZP values among the liposomes formulated at different FRRs. Regardless of the different microfluidic chips used for formulation, the produced liposomes exhibited a common pattern in vesicle size on day 0. In the current study, DSPC liposomes were observed to be smaller than DMPC liposomes on day 0. The data of the literature in this area are inconsistent. According to Zook and Vreeland [23], DMPC liposomes are considered to exhibit more elasticity in their bilayer membrane and thus have are smaller than DSPC liposomes, which tend to have a more rigid bilayer membrane due to their longer alkyl chain (18:0 PC) compared to DMPC (14:0 PC). However, the current study’s findings align with the study of Ekonomou et al. [6]. The results of the present study are also in agreement with the data of Perrie et al. [24], where DMPC liposomes formed at FRR 2:1 were larger than DSPC liposomes. This observation suggests that an increase in alkyl chain length is associated with a decrease in the size of liposomes formed [25]. However, it is important to consider the difference in the liposome-enclosed compounds in the above-mentioned studies. When comparing the size of liposomes prepared with the same lipid, but different microfluidic chips, the differences observed were small, but statistically significant (Figure 1 and Figure 2A,E, *p* < 0.05). DMPC and DSPC liposomes at FRR 2:1 produced using the serpentine pattern chip showed no significant differences when compared to the same liposomes made with the Y-shaped chip (Figure 1 and Figure 2, *p* > 0.05). The variation in the size and PDI of liposomes from different microfluidic chips can be attributed to the difference in the micromixing channel designs. Micromixing channels with different designs impact the contact area and association time between the ethanol and aqueous phase during the course of the mixing process. Additionally, the length of the mixing channels affects the diffusion of the two solvents [8]. All these factors can contribute towards liposomes with different physical parameters, such as size and PDI. The length of the main mixing channels has been shown to affect the mixing of the ethanol and aqueous phases. Curves in the serpentine microfluidic chip facilitate efficient mixing, with complete mixing of solvents at different FRRs [26].

With regard to PDI, variations among DMPC liposomes (FRR 4:1 and 5:1) from different chips were again small but statistically significant (Figure S2C,G, *p* < 0.05). It is interesting to note that DSPC liposomes (FRR 2:1, 3:1, 4:1, 5:1) from both the microfluidic chips did not show any significant difference in PDI (Figure S2, *p* > 0.05).

Microfluidics has an advantage because it permits the synthesis of liposomes with a low volume of solvents compared to conventional methods, which utilise larger volumes of solvents to prepare EO-loaded liposomes [10,27]. In addition, liposome production is more time-efficient due to a high degree of automation, which increases the reproducibility of the results. This is clearly evidenced in the present study, where liposomes from different batches displayed minimal variations in parameters such as size, PDI and ZP.

### 3.3. Effect of Storage Temperature on Liposomes’ Stability

For liposomes to be an efficient delivery system, their stability must be retained for the intended storage period. Evaluating the stability of liposomes is also crucial for providing information about the retention and release of the enclosed compounds. This is particularly relevant to designing the dosage of enclosed compounds in food production. As the temperature is a vital factor that can affect the physiochemical properties of the liposomes [23], in this study, the eugenol-encapsulated liposomes were incubated at three different temperatures for 60 days. The stability of liposomes was assessed by measuring physicochemical parameters such as size, PDI, and (ZP) over this 60-day period. Figure 1 and Figure 2 show the mean size of liposomes throughout the storage period at different temperatures (4, 20, and 37 °C), produced using serpentine and Y-shaped microfluidic chips, respectively. The mean size of liposomes on day 0 made using the serpentine microfluidic chip was in the range of 117.5 nm to 189.3 nm, and variation in size by the end of the storage period ranged from 121.16 nm to 215.76 nm (Figure 1). In the case of liposomes produced with the Y-shaped microfluidic chip, the mean size on day 0 was between 113.33 and 176.96 nm, and the changes by the end of the storage period ranged from 105.30 nm to 345.26 nm (Figure 2).

The most stable liposomal formulation with negligible changes in the physicochemical parameters (size, PDI, and ZP) during the storage at 4, 20, and 37 °C were DSPC FRR 5:1 liposomes produced with the serpentine chip (Figure 1D). There was a slight, but not statistically different increase in the size after 14 days, after which no change was observed till the end of storage of period. DSPC serpentine formulations (Figure 1A–D, *p* > 0.05) were stable throughout the storage period at all temperatures. This is in alignment with results obtained by Peng et al. [28], who observed eugenol liposomes to be stable for eight weeks with minor changes in size when stored at 4℃ and room temperature. In the case of DMPC serpentine liposomes, most fluctuations in size were observed at 37 °C, especially for FRR 2:1, which showed a statistically significant decrease in size over the storage period (Figure 1E, *p* < 0.05). This was accompanied by a statistically significant increase in PDI on day 14, after which it remained constant till the end of the storage period (Figure S2E, *p* < 0.05). This is likely due to the liposomal formulation establishing a geometrical state of low entropy at this time point, explaining the plateau in PDI change after this time. A clear trend was observed for DMPC FRR 2:1 liposomes produced with the Y-shaped microfluidic chip, where an increase in the size of liposomes was analogous to the rise in PDI, as seen by Lu et al. [29]. For liposomes stored at 37 °C (Figure 2E), a decrease in size was associated with an increase in PDI (Figure S3E). However, that was not an established trend, and other liposomes, e.g., DSPC FRR 2:1 liposomes (Figure 2A), DSPC FRR 3:1 (Figure 2C) prepared the using serpentine chip and DMPC FRR 2:1 (Figure 2E) and DMPC FRR 3:1 (Figure 2F) prepared using the Y-shaped chip showed an increase in size and PDI (Figure S3A,C,E,F) at 4 and 20 °C during storage. Amongst the liposomes prepared using the Y-shaped chip, DSPC FRR 5:1 (Figure 2D) was the most stable, with no significant changes in size or PDI (Figure S3D, *p* < 0.05).

Regarding size, most of the liposomes were stable at 4 and 20 °C compared to 37 °C showing slight variations, which coincides with the results reported by other recent studies [20,30]. In contrast, DSPC liposomes were found to be more stable during storage than DMPC liposomes. DSPC liposomes showed greater stability with regard to size when stored at 20 and 37 °C for 30 days [6]. This can be attributed to the difference in transition temperature (T_c_) of these lipids. DMPC’s transition temperature (Tc) is at 24 °C. Therefore, storage of DMPC liposomes at 37 °C affects the gel-to-liquid transition, triggering a change in the arrangement of the fatty acid chain [7], whereas T_c_ of DSPC is 55 °C, allowing this lipid to retain its gel state at 37 °C.

The decrease in the particle size of the liposomes at different temperatures can be due to the leakage of the enclosed compound over time [6]. Weaver et al. [30] suggested that this effect could be due to the diffusion of the enclosed compound to the area of lower concentration led by osmosis. An increase in temperature can disturb the hydrogen bonds between the phospholipids, making the bilayer more fluid and altering liposomes’ size [31]. Furthermore, the increase in the diameter of liposomes can also be attributed to phenomena such as aggregation and flocculation. This aggregation can be induced by electrostatic attraction between the liposomes, which is governed by the charge present on the surface or distortion of the lipid bilayer [32].

With regard to PDI, small but significantly different fluctuations were observed during storage at 4 and 37 °C (*p* < 0.05), and all the values were below 0.250, indicating a monodispersed liposomal formulation after 60 days of storage, except for DSPC 2:1, DSPC 3:1, DMPC 2:1, and DMPC 3:1 prepared with the Y-shaped chip (Figure S3A,B,E,F). According to the literature, there is an increase in PDI of liposomes stored at temperatures higher than 20 °C, which can be attributed to the high-temperature-mediated coalescence of liposomes [20]. At the same time, other authors showed an increase in PDI of liposomes with enclosed thyme oil at 4 and 20 °C during the storage [10], which is consistent with the results of the present study.

A range of factors such as pH, temperature, ionic strength, solvent viscosity, phospholipid composition and compound enclosed can influence ZP. ZP values within −25 to +25 mV signify stable liposomes [6]. Repulsion caused by the same charges will prevent clustering or coagulation, resulting in a stable colloidal dispersion. Liposomes in the present study exhibited ZP in the range of −17 to −30 mV at all temperatures during 60 days of storage, with a few exceptions.

Diminished size and PDI are desirable characteristics for liposomes to be used in food products. In the present study, at the end of the storage period (day 60), the size of the liposomes was below 300 nm. Small liposomes have a larger surface area, thus enhancing the interaction with microorganisms and augmenting the antimicrobial effect. Increased surface area facilitates higher encapsulation of antimicrobial compounds and consequently, the higher release of the enclosed compound into the food product. Smaller liposomes are easy to diffuse, and homogeneous distribution into the food matrix can be achieved [33]. The PDI value of the liposomes on day 60 was below 0.3, indicating a homogeneous distribution of liposomes in the formulations. This will ensure the uniform release of the enclosed bioactive compound in the food, making these suitable to be used as antimicrobial food-grade liposomes. An optimum range for ZP for liposomes is not provided in the literature. However, in this study, liposomes showed minimal aggregation (based on the DLS measurements), indicating the presence of the same charge on their surface. Overall, comparing the liposome physicochemical parameters at the beginning and end of the storage period, most liposomal formulations were stable for 60 days at the tested storage temperatures (4, 20, and 37 °C).

### 3.4. FTIR Spectra Acquisition

**Figure 3:**
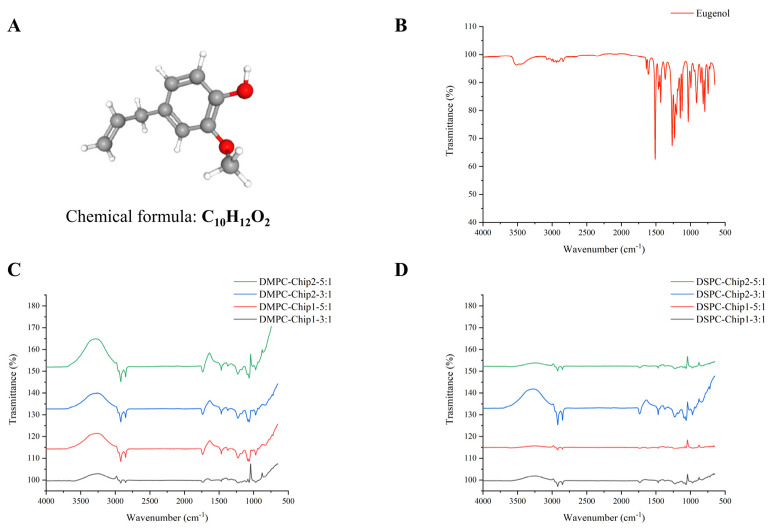
(**A**) Chemical structure of eugenol (adapted from PubChem [36]). FTIR spectra obtained using (**B**) the investigated antimicrobial compound eugenol, (**C**) DMPC, and (**D**) DSPC eugenol-loaded liposomes produced at 3:1 and 5:1 FRR, using both microfluidic chips with a serpentine and Y-shaped pattern.

FTIR analysis was performed for pure eugenol alone and the most suitable DMPC and DSPC liposomal formulations at FRR 3:1 and 5:1 to investigate the chemical bonds within the samples (Figure 3). Before encapsulation, FTIR spectra of eugenol revealed two characteristic peaks responsible for the stretching vibrations of −OH groups at 3515 and 3457 cm^−1^, the C–H stretching vibration peaks at 2700–3000 cm^−1^, and the peaks at 1500–1600 cm^−1^ and close to 1300 cm^−1^ responsible for the C=C aromatic groups of our compound (Figure 3A). The lack of those signals (at 3515 and 3457 cm^−1^) accountable for the stretching vibrations of −OH groups in eugenol on FTIR spectra and the disappearance of the signals revealing the stretching vibrations of C-O near 1100 cm^−1^ confirmed the encapsulation of eugenol during the preparation of the DMPC and DSPC liposomal formulations (Figure 3B,C). The observed frequencies are consistent with data from the literature [34,35]. The phospholipids revealed similar absorption peaks and chemical bonds, including the (O–H) bond of alcohol peaks in the range 3209–3348 cm^−1^. Finally, as observed in our previous study, the vibrations of PO4^−^ phosphate head groups detected at 1052–1126 cm^−1^ indicate symmetric stretching vibrations, revealing the formulation of both DMPC and DSPC liposomes [6].

### 3.5. AFM-Based Characterisation

**Figure 4:**
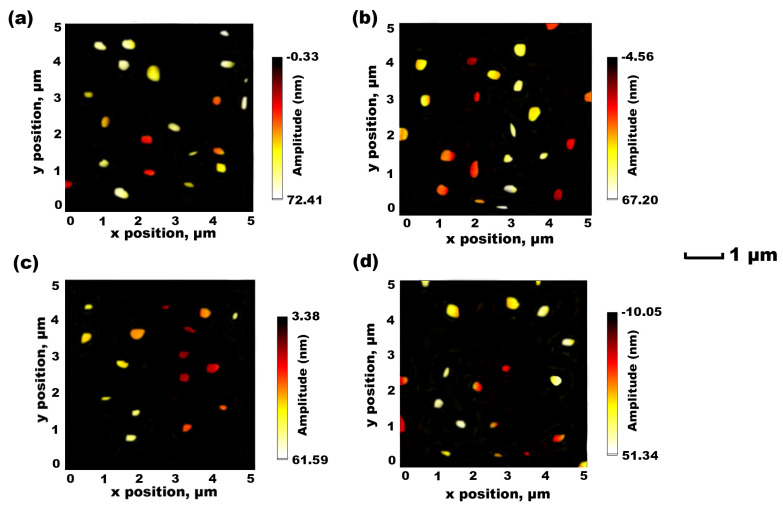
AFM images obtained of eugenol-loaded DMPC liposomes, produced at 3:1 FRR, using a microfluidic chip (**a**) with a serpentine pattern and (**b**) a Y-shaped pattern and eugenol-loaded DSPC liposomes, produced at 3:1 FRR, using a microfluidic chip (**c**) with a serpentine pattern and (**d**) a Y-shaped pattern.

Using different types of lipids (DSPC and DMPC) for liposome fabrication through different FRR and microfluidic mixing patterns (serpentine and Y-shaped) provides liposomes with different diameters (Figure 4). In the current study, the most suitable formulations for DMPC and DSPC lipids were produced through TFR 0.50 mL min^−1^ at an FRR of 3:1. The AFM images in Figure 4 represent the morphological shape of DMPC and DSPC liposomes after encapsulation of eugenol. The DMPC liposomes prepared using both microfluidic chips showed a uniform distribution and compressed or flattened spheroidal shapes [6,30]. The DSPC liposomes loaded with eugenol and presented in Figure 4c,d were the smallest with higher shape uniformity than the DMPC liposomes in Figure 4a,b. When the DMPC and DSPC liposomes were produced using a microfluidic chip with a Y-shaped pattern, their morphological characteristics changed and became more circular and regular (Figure 4b,d). In previous AFM imaging performed by Weaver et al. [30] using a Y-shaped microfluidic chip pattern, very similar shapes of rounded bodies of phospholipids were observed. The AFM results confirm that the average particle size was similar to the size measured by DLS and less than 200 nm for DMPC liposomes and 150 nm for DSPC liposomes. Slight variations in the diameter, shape, and size might be due to the effect of the drying process used for liposome production [6,20].

### 3.6. Encapsulation Efficiency

**Figure 5:**
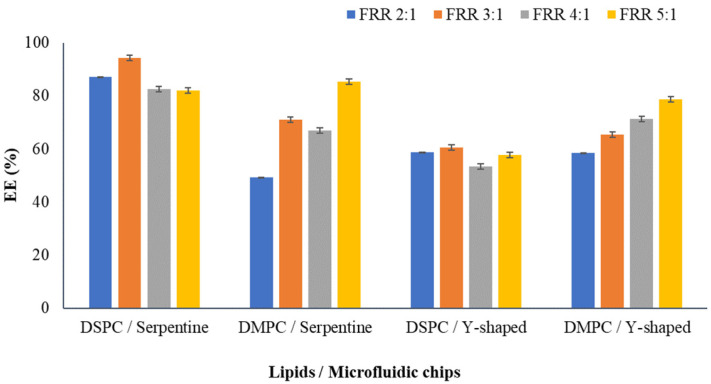
EE (%) of eugenol-enclosed DSPC and DMPC liposomes produced with serpentine and Y-shaped microfluidic chips.

The encapsulation efficiency (EE) of eugenol in DSPC and DMPC liposomes was measured by UV-vis spectrophotometry. Higher EE ensures a higher concentration of bioactive compounds delivered to the food products. The encapsulation efficiency of different liposomes is presented in Figure 5. EE values ranged from 49.23% to 94.34%. All liposomal formulations showed high EE, and the highest value of 94.34% was noted for the DSPC FRR 3:1 prepared using a microfluidic chip with a serpentine micromixing design (Figure 5). In the case of the liposomes prepared using a Y-shaped chip, the highest EE of 78.63% was observed for DMPC FRR 5:1 liposomes (Figure 5). It can be concluded that the EE of eugenol-loaded liposomes produced using microfluidics is higher than liposomes enclosing eugenol produced by the thin-film hydration method, which revealed an EE of only 35.0% [29]. DSPC liposomes made with the serpentine chip showed high EE compared to DMPC liposomes, in accordance with the results reported by Ekonomou et al. [6]. Similar results were obtained in the study of Xu et al. [37], which revealed a higher EE of proteins within DSPC liposomes. The difference in the EE between the liposomes can be attributed to the alkyl chain length of the lipids and their transition temperatures. DSPC is saturated with a longer alkyl chain, leading to stronger chain–chain interaction and resulting in a closely packed structure and increased spatial area [38]. Though the EE was higher for DSPC liposomes from the serpentine chip, with regard to the Y-shaped chip, DMPC liposomes showed higher EE. It is important to note that in the case of Y-shaped DMPC liposomes, an increase in FRR resulted in an increase in EE from 58.52% at FRR 2:1 to 78.64% at FRR 5:1 (Figure 5). According to the literature, DMPC liposomes present high EE values, i.e., 75% for phenolic compounds [39]. The EE of liposomes is mainly dependent on the type and physicochemical properties of the enclosed compound, the method of liposome preparation, the type of lipid used, the length of fatty acid chain, the degree of saturation, the concentration of cholesterol and the ratio of the proportion of lipid to cholesterol [40].

### 3.7. Eugenol Release Analysis

**Figure 6:**
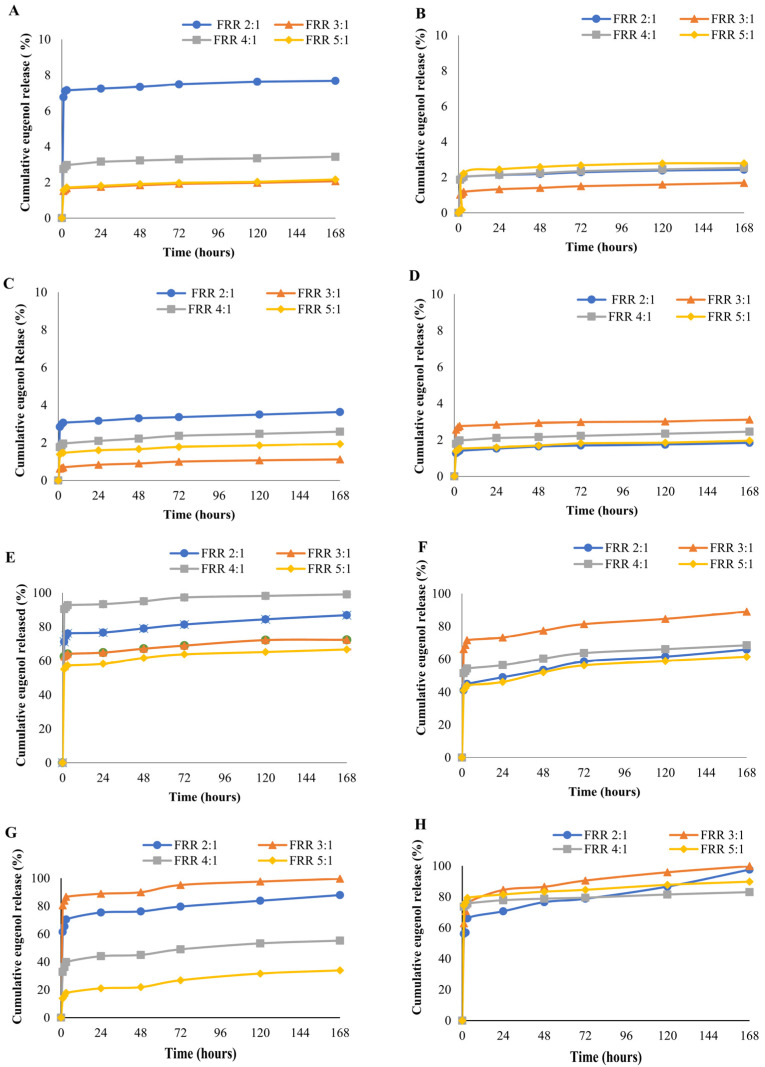
Cumulative eugenol release (%) from liposomes at pH 7.4: (**A**) serpentine DSPC; (**B**) serpentine DMPC; (**C**) Y-shaped DSPC; (**D**) Y-shaped DMPC at pH 4.0; (**E**) serpentine DSPC; (**F**) serpentine DMPC; (**G**) Y-shaped DSPC; (**H**) Y-shaped DMPC.

The controlled release of the enclosed compound in liposomal formulations allows for increasing the compounds’ bioavailability. Furthermore, encapsulation of the compounds in liposomes increases their stability, as the compounds are protected from the external environment [41]. In the present study, in vitro eugenol release was investigated at two common food pH values of 7.4 and 4.0, and the results are presented in Figure 6.

A drastic difference was observed in the drug release pattern from different liposomes under these pH conditions. At pH 7.4, the total eugenol release was in the range of 7.68% to 1.11% from the liposomes produced with the serpentine chip (Figure 6). This high retention of eugenol can be attributed to the stability of the liposomes at pH 7.4. The highest eugenol release of 7.68% and 3.63% was observed for the DSPC FRR 2:1 liposomes produced with the serpentine and Y-shaped chips, respectively (Figure 6A,C). At pH 7.4, DSPC FRR 3:1 and 5:1 produced with the serpentine chip, no significant differences were observed in the eugenol release throughout the 7 days of storage period (Figure 6A, *p* > 0.05). In the case of DMPC FRR 2:1 and FRR 4:1 liposomes from the same chip, though the eugenol release seemed to be overlapping, it was significantly different (Figure 6B, *p* < 0.05), except for 2 and 3 h, where no significant differences were observed (Figure 6B, *p* < 0.05). DSPC liposomes from both the microfluidic chips (Figure 6A,C,E,G) exhibited a higher eugenol release than DMPC liposomes at pH 7.4, with some exceptions. FRR 3:1 DMPC liposomes with the Y-shaped chip (Figure 6D) displayed the higher eugenol release than FRR 3:1 DSPC liposomes (Figure 6C, *p* < 0.05). During the 7-day incubation period, the level of eugenol revealed a significant increase in the first 3 h and then remained constant. Its release remained stable throughout the entire period, with a slight increase towards the end. Data from the literature suggest that the permeability of DSPC liposomes can be increased by the presence of cholesterol [42]. However, in the current study, both DMPC and DSPC liposomes were formulated using a lipid-to-cholesterol ratio of 2:1. Therefore, it is unlikely that the presence of cholesterol is the cause of the higher drug release from DSPC liposomes. Meher and Chakraborty [43] reported that eugenol’s interaction with DMPC can increase the gel phase of DMPC. This might be the reason for the lower drug release from DMPC when compared to DSPC liposomes at pH 7.4.

A higher eugenol release was observed for all liposomes at pH 4.0 compared to pH 7.4 (Figure 6E–H). DSPC liposomes produced with the serpentine chip (Figure 6E) showed significantly higher eugenol release ranging from 66.68% to 99.12% compared to DMPC liposomes, which ranged from 61.38% to 88.96% (Figure 6F, *p* < 0.05). This was similar to the pattern observed for drug release at pH 7.4. The highest eugenol release (99.12%) was observed from DSPC FRR 4:1 liposomes produced with the serpentine chip, where most of the eugenol (93.36%) was released in the initial 24 h of incubation. Delama et al. [44] also reported lower iohexol release from DMPC liposomes than DSPC liposomes.

In contrast, DMPC liposomes produced with the Y-shaped chip showed a significantly higher eugenol release (between 83.04% and 99.86%) (Figure 6H) than that from DSPC liposomes (between 33.91% and 99.67%) (Figure 6G). The highest eugenol release at pH 4.0 was observed from the liposomes manufactured using the Y-shaped chip at FRR 3:1 (Figure 6F–H). Furthermore, at this pH value, a burst release of eugenol was observed in the first three hours of incubation. This was followed by a further significant increase in eugenol release up to 72 h. A similar eugenol release profile was observed for all the liposomes from both microfluidic chips. These results are in agreement with the data reported by Ekonomou et al. [6].

Change in the pH can alter the protonated or deprotonated state of the functional groups present in the bilayer lipids, causing morphological changes and ultimately triggering a change in the permeability of the liposomes [45]. This can be one of the factors that caused the higher eugenol release at pH 4.0 compared to pH 7.4 from the liposomes produced in the current study.

## 4. Conclusions

In this study, we investigated the encapsulation of eugenol, a natural antimicrobial compound, in liposomes. Our results demonstrate that eugenol exhibits promising antimicrobial properties against *E. coli* and *S. aureus*, making it suitable for use in food systems. We employed microfluidics to produce liposomes encapsulating eugenol, which were characterised by their small size, homogeneity, PDI, and reproducibility. Additionally, we identified specific microfluidic manufacturing conditions that enabled the production of stable eugenol-enclosed liposomes that remained stable across a range of temperatures during storage. Furthermore, the liposomes produced in this study exhibited high EE and demonstrated different eugenol release patterns at various pH levels, which opens possibilities for further exploration in acidic and neutral pH food environments. Overall, these liposomal formulations exhibit significant potential as food additives for pathogen control and enhanced food safety.

## Data Availability

Data is contained within the article and Supplementary Materials.

## Supplementary Materials

The following supporting information can be downloaded at https://www.mdpi.com/article/10.3390/foods12152940/s1. Figure S1. Microfluidic chips with serpentine (left) and Y-shaped (right) micromixing channels. Figure S2: PDI of eugenol-loaded liposome during storage. (A) Serpentine DSPC FRR2:1; (B) serpentine DSPC 3:1; (C) serpentine DSPC 4:1; (D) serpentine DSPC 5:1; (E) serpentine DMPC 2:1; (F) serpentine DMPC 3:1; (G) serpentine DMPC 4:1; (H) serpentine DMPC 5:1. Uppercase letters denote the difference in the size of liposomes for a single temperature on different days. Lowercase letters denote the difference in the size of liposomes at different temperatures on the same day. Figure S3: PDI of eugenol-loaded liposome during storage. (A) Y-shaped DSPC FRR2:1; (B) Y DSPC 3:1; (C) Y DSPC 4:1; (D) Y DSPC 5:1; (E) Y DMPC 2:1; (F) Y DMPC 3:1; (G) Y DMPC 4:1; (H) Y DMPC 5:1. Different uppercase letters denote the difference in the size of liposomes for a single temperature on different days. Different lowercase letters denote the difference in the size of liposomes at different temperatures on the same day.
