# Supplementary material for: The Manufacturing and Characterisation of Eugenol-Enclosed Liposomes Produced by Microfluidic Method
Source: Foods. 2023 Aug 3;12(15):2940. doi: 10.3390/foods12152940 (PMC10418319; doi:10.3390/foods12152940)
Supplement: Supplementary file 1 [file foods-12-02940-s001.zip › foods-2522161-supplementary.pdf]

Article

The manufacturing and characterisation of eugenol-enclosed liposomes produced by microfluidics method

Jessica Ghodke[1], Sotirios I. Ekonomou[1], Edward Weaver[2], Dimitrios Lamprou[2], Olena Doran[1], Alexandros Ch. Stratakos[1]*

1 College of Health, Science and Society, University of the West of England, Coldharbour Ln, Bristol, BS16 1QY, UK.; Jess.Ghodke@uwe.ac.uk (J. G.); sotirios.oikonomou@uwe.ac.uk (S. E.); Olena.Doran@uwe.ac.uk (O.D.); alexandros.stratakos@uwe.ac.uk (A. S.)

2 School of Pharmacy, Queen's University Belfast, 97 Lisburn Road, Belfast BT9 7BL, UK.; D.Lamprou@qub.ac.uk (D. L.); eweaver01@qub.ac.uk (E. W.)

* Correspondence: alexandros.stratakos@uwe.ac.uk

Supplementary material

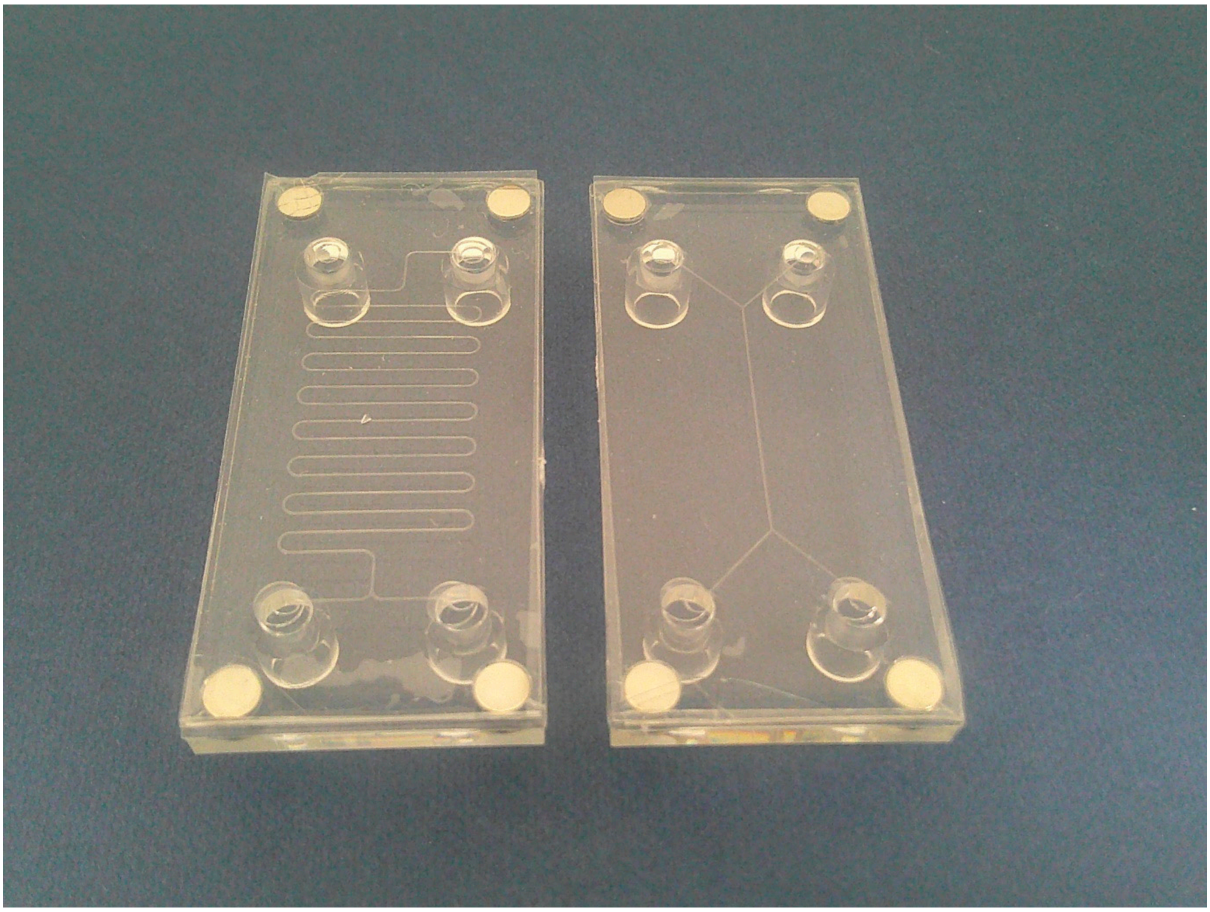

Figure S1. Microfluidic chips with serpentine (left) and Y-shaped (right) micromixing channels.

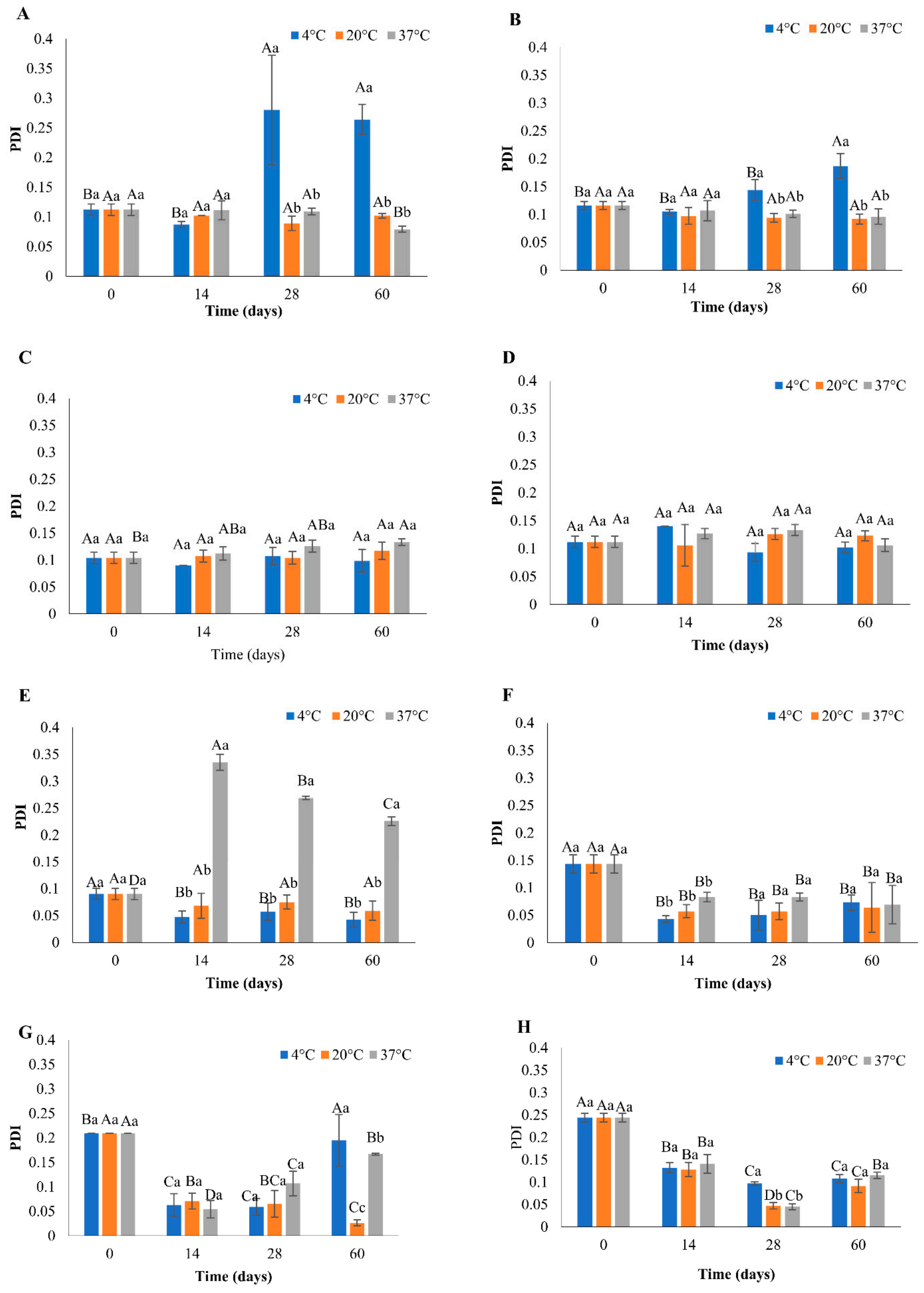

Figure S2. PDI of eugenol-loaded liposomes during storage: (A) serpentine DSPC FRR2:1; (B) serpentine DSPC 3:1; (C) serpentine DSPC 4:1; (D) serpentine DSPC 5:1; (E) serpentine

DMPC 2:1; (F) serpentine DMPC 3:1; (G) serpentine DMPC 4:1; (H) serpentine DMPC 5:1. Different uppercase letters denote a difference in the size of liposomes for a single temperature on different days. Different lowercase letters denote a difference in the size of liposomes at different temperatures on the same day.

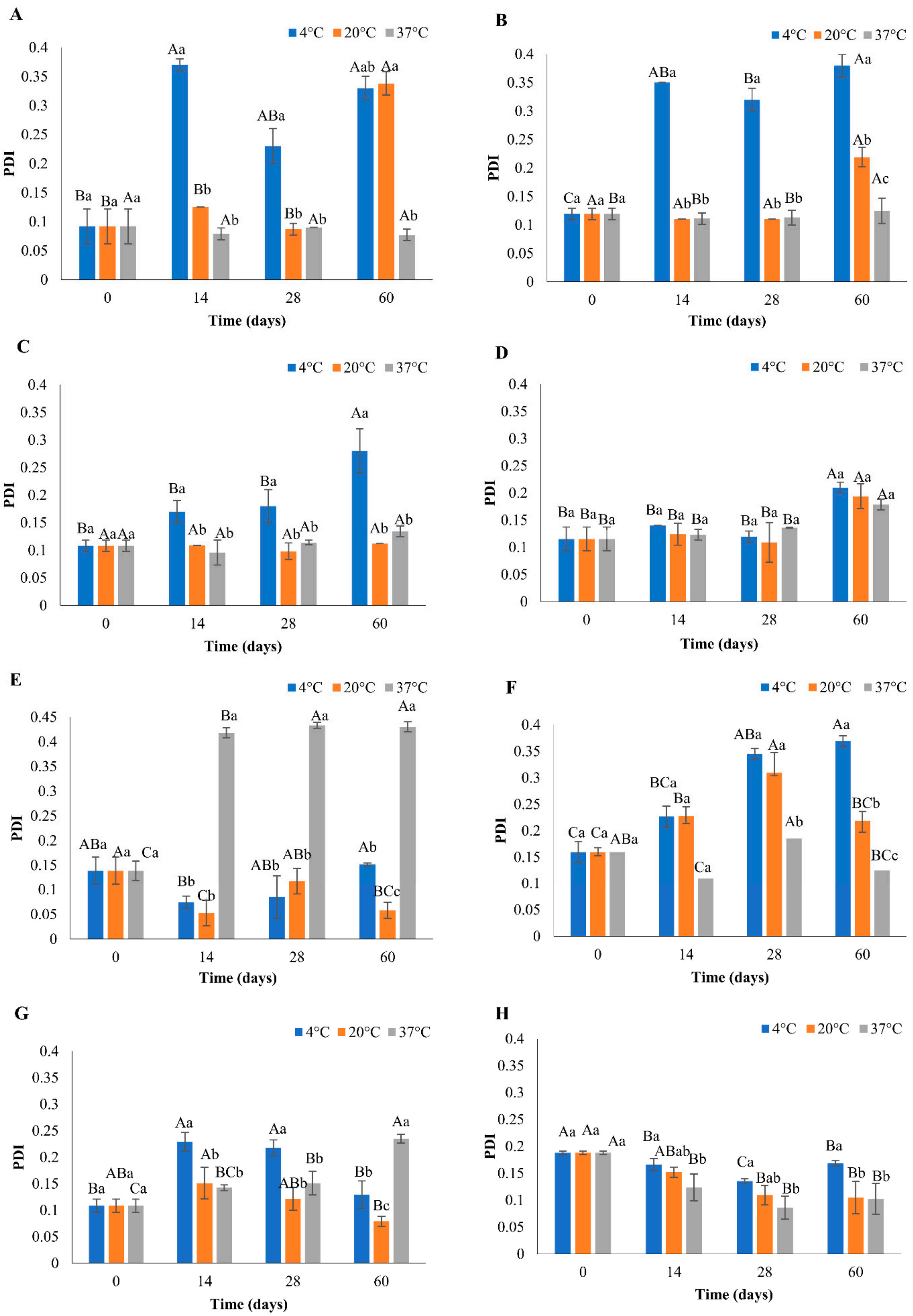
A
B
C
D
E
F
G
H
4°C
20°C
37°C
PDI
Time (days)
0
14
28
60

Figure S3. PDI of eugenol-loaded liposomes during storage: (A) Y-shaped DSPC FRR2:1; (B) Y DSPC 3:1; (C) Y DSPC 4:1; (D) Y DSPC 5:1; (E) Y DMPC 2:1; (F) Y DMPC 3:1; (G) Y DMPC 4:1; (H) Y DMPC 5:1. Different uppercase letters denote a difference in the size of liposomes for a single temperature on different days. Different lowercase letters denote a difference in the size of liposomes at different temperatures on the same day.
